# Towards a Diagnostic Tool for Diagnosing Joint Pathologies: Supervised Learning of Acoustic Emission Signals

**DOI:** 10.3390/s21238091

**Published:** 2021-12-03

**Authors:** Khadijat A. Olorunlambe, Zhe Hua, Duncan E. T. Shepherd, Karl D. Dearn

**Affiliations:** 1 Mechanical Innovation and Tribology Group, Department of Mechanical Engineering, School of Engineering, University of Birmingham, Birmingham B15 2TT, UK; KAO863@student.bham.ac.uk (K.A.O.); 17121266@bjtu.edu.cn (Z.H.); 2 Biomedical Engineering Group, Department of Mechanical Engineering, School of Engineering, University of Birmingham, Birmingham B15 2TT, UK; d.e.shepherd@bham.ac.uk

**Keywords:** acoustic emission, wear, k-means clustering, neural networks, supervised learning

## Abstract

Acoustic emission (AE) testing detects the onset and progression of mechanical flaws. AE as a diagnostic tool is gaining traction for providing a tribological assessment of human joints and orthopaedic implants. There is potential for using AE as a tool for diagnosing joint pathologies such as osteoarthritis and implant failure, but the signal analysis must differentiate between wear mechanisms—a challenging problem! In this study, we use supervised learning to classify AE signals from adhesive and abrasive wear under controlled joint conditions. Uncorrelated AE features were derived using principal component analysis and classified using three methods, logistic regression, k-nearest neighbours (KNN), and back propagation (BP) neural network. The BP network performed best, with a classification accuracy of 98%, representing an exciting development for the clustering and supervised classification of AE signals as a bio-tribological diagnostic tool.

## 1. Introduction

Tribological interactions are fundamental to the operation of artificial joints, and wear is a principal means of failure in these devices [1,2]. The three most common forms of wear by which artificial joints fail are adhesive, abrasive, and fatigue wear [3,4,5]. Artificial joint failures are traditionally diagnosed using X-rays and CT (computed tomography) scans, methods that are expensive, time-consuming, and harmful to health due to frequent radiation exposure [6,7]. There is also a concern with these traditional diagnostic techniques that signs of failure may not present early enough to prevent pathologies, causing patients to experience pain and allowing the migration of wear debris into the bloodstream, leading to further medical complications [1,6,8]. Thus, there is a need for a simplified and faster way of diagnosing artificial joint failures to avoid these problems, and this is where the application of acoustic emission (AE) testing has potential.

AE testing is a non-destructive test (NDT) to detect mechanical flaws’ onset and progression [8,9]. In addition, AE testing has proven helpful in the tribological studies of human joints [10,11,12,13] and the condition monitoring of artificial joints [14,15,16]. AE technology’s recent progress and development suggest a potential to diagnose joint pathologies such as osteoarthritis and artificial joint failure based on the differentiation between wear mechanisms. One of the difficulties in achieving this diagnostic potential of AE testing is processing large data sets generated during data acquisition. However, machine learning techniques such as clustering, principal component analysis (unsupervised learning), and neural net classification (supervised learning) provide a means to overcome this problem. Gutkin et al. [17] analysed AE signals from carbon fibre-reinforced polymers under various test configurations using three pattern recognition algorithms: k-means clustering, Self-Organising Map (SOM-k), combined with k-means and Convolutional Neural Network (CNN). SOM-k was the most effective at classifying AE responses to failures. Qiao et al. [18] also used k-means clustering to classify AE signals from ceramic thermal barrier coatings during indentation testing into three distinct categories associated with different failure modes. Using back propagation (BP) neural networks to further identify failure types of the coatings after thermal exposure, the results showed that AE measurements could distinguish between the mechanisms of high-temperature oxidation that accelerated thermal barrier degradation. Yao et al. [19] used a combination of the wavelet fuzzy neural network with AE and fuzzy classification combined with a motor current to estimate tool wear successfully. Machine learning approaches, combined with AE, enable a deeper categorisation of signals based on damage recognition and failure modes. These techniques should provide a platform to diagnose orthopaedic pathologies and implant failures early and identify causes.

This paper reports how machine learning techniques classify AE signals according to wear mechanisms (i.e., adhesive and abrasive) initiated under controlled joint conditions using supervised and unsupervised learning methods. In the context of this study, adhesive wear in a smooth polymer–metal counterface occurs when wear polymer particles adhere to the metal surface during sliding. Abrasive wear occurs when the smooth polymer is sliding on a hard and rough metal surface, thereby causing material removal and a possible three-body abrasive wear.

To date, analysis of AE in orthopaedics application has focused chiefly on parameter and frequency-based techniques [13,14,15,16,17]. Focusing only on these techniques can be computationally expensive and time-consuming as it relies heavily on the user’s knowledge of the influence of the different derived parameters to find significant relationships for interpretation. Machine learning techniques as a pattern recognition tool can discover the relationship between AE signals and the underlying tribological behaviour. The study by Shark et al. [20] shows that AE can differentiate between healthy knees and osteoarthritic knees using basic analysis techniques. In a further study [13] the authors applied the principal component analysis technique to knee AE feature profiles. They found that osteoarthritic knees could be further grouped based on age and progression of arthritis, showing the advantage of applying pattern recognition techniques. Through machine learning methods, analysis of AE signals could be more robust to diagnose specific tribological mechanisms occurring in an artificial joint. The machine learning algorithms need to be trained to recognise the specific mechanisms that have been identified [2,21,22,23,24] to be related to the different failure modes (predominantly adhesive and abrasive wear) of artificial joints, which is the aim of this paper.

Analysing AE signals using a combination of machine learning methods defined in the above paragraphs and the standard techniques provides a deeper understanding of ortho-tribological phenomena, providing a pathway towards developing a rapid diagnostic tool for joint pathologies.

## 2. Materials and Methods

### 2.1. Experimental Parameters

**Figure 1:**
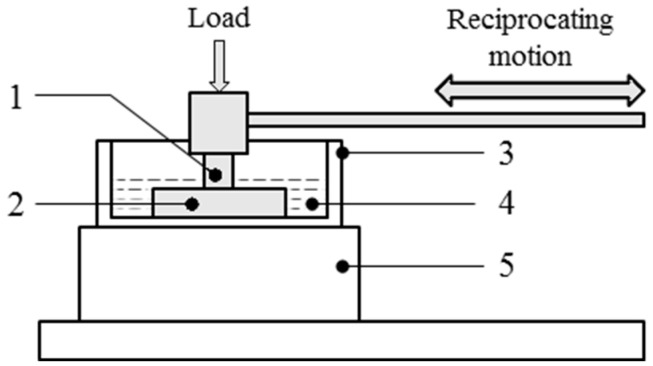
Experimental layout of TE77 tribometer. (1) PEEK upper specimen and mounted AE sensor, (2) steel lower specimen, (3) lubricant bath, (4) lubricant (ringer’s solution), (5) heater block.

AE signals were acquired using a tribo-acoustic test set-up consisting of a tribometer and an AE acquisition system, with parameters selected to simulate abrasive and adhesive wear tests. The experiment layout is shown in Figure 1. Reciprocating sliding tests were performed on a TE77 High-Frequency Reciprocating Machine (Phoenix Tribology, Newbury, UK) using a cylinder-on-plate configuration (representing line contact). Tests were performed using poly-ether-ether-ketone (PEEK) rods (6 mm diameter, 16 mm length, supplied by Direct Plastics, Sheffield, UK) as the reciprocating specimen and steel plates as the fixed lower-specimen (dimensions shown in Figure 1). The materials were chosen to represent a metal-on-polymer joint replacement articulation. The PEEK rods were cleaned before and after each test following the method described in ASTM F732-17 [25]. In addition, the steel plates were washed in ethanol before and after each test. Before cleaning and testing, the steel plates were roughened to simulate abrasive wear using a belt sander fitted with P40 grade sandpaper. Tests ran for 1 h and at 2 Hz frequency. A quarter strength Ringer’s solution (1 tablet dissolved in 500 mL distilled water) was used as a lubricant for the abrasive tests. In order to generate enough AE data for classification, there were three runs for each wear mechanism. The wear scars were captured using the Alicona Infinite Focus (Alicona Imaging Gmbh, Graz, Austria), an optical 3D surface measurement system. The images were captured at a magnification of 20× with the polariser turned off.

#### 2.1.1. Test Parameters

Hertzian contact mechanics [26] were used to calculate the initial test load, based on the contact conditions typically found in a ball-and-socket Charite Lumbar Spinal Implant, with 10 mm ball radius and 0.35 mm radial clearance [27], and loading and displacement conditions defined by BS ISO 18192-1 for wear of total intervertebral spinal disc prostheses [28]. Under worst-case conditions, the calculated load and sliding velocity are given in Table 1.

#### 2.1.2. AE Signal Acquisition and Post-Processing

AE signals were acquired using an acquisition and recording system (supplied by the Mistras Group, Cambridge, UK) comprising a nano-30 AE sensor, a 2/4/6 preamplifier with a gain of 60 dB, and the AEWin PCI2 software. The sensor was fixed to the upper specimen holder (Figure 1, item (1)), which was connected to the preamplifier and then to a computer with the software installed for signal conditioning and acquisition.

Signals were acquired at a sampling frequency of 2 MHz throughout the tests. The acquired AE signals were then post-processed using the NOESIS Advanced AE Analysis software from Mistras Group to derive new discrete AE features such as amplitude and duration. Further analyses were carried out using MATLAB. Selected features were collated and loaded into MATLAB (R.2019a) for analysis using pattern recognition techniques from the machine learning and deep learning toolbox and outlined in Section 2.2 below.

### 2.2. Pattern Recognition Techniques

The portfolio of pattern recognition techniques used to drive the learning and matching between recorded AE data and the information stored in the training database is outlined below.

#### 2.2.1. Feature Selection and Extraction

AE features for clustering and classification were selected using hierarchical link clustering and principal component analysis (PCA). AE waveforms were defined using discrete parameters/features to allow pattern recognition techniques based on multi-parameter statistical analysis. However, post-processing of the AE signals produced 50+ AE features. Therefore, the input AE features were minimised to increase the speed and accuracy of classification. The feature selection method used is described in [29]. First, the ten most common features used in previous studies were selected [18,30,31] and then normalised to give a weighting using $X^{\prime }=\frac{\;X-\mu }{\sigma }$ with μ the mean value of the descriptor and σ the standard deviation. Next, the correlation matrix was calculated and subjected to complete link hierarchical clustering. Eight features with Pearson correlation coefficients in the range [−0.7:0.7] were then selected. The eight selected features are shown in Table 2.

PCA enabled feature extraction for the supervised classification of the signals

Using PCA, new uncorrelated features were determined by linear combinations of the features selected for clustering purposes. The principal components were chosen using the process outlined below:

Normalised AE features were composed into a matrix X of dimensions *n* by *m* (Equation (1)):

(1) $$X=[\begin{array}{cccc} x_{11} & x_{12} & \cdots & x_{1m}\\ x_{21} & x_{22} & \cdots & x_{2m}\\ \vdots & \vdots & \ddots & \vdots \\ x_{n1} & x_{n2} & \cdots & x_{nm} \end{array}]$$

The eigenvalues of the covariance matrix and the corresponding eigenvectors were then obtained (Equations (2) and (3)).

(2) $$R=Cov\left(X\right)=\left[\begin{array}{cccc} r_{11} & r_{12} & \cdots & r_{k1}\\ r_{21} & r_{22} & \cdots & r_{2k}\\ \vdots & \vdots & \ddots & \vdots \\ r_{k1} & r_{2k} & \cdots & r_{kk} \end{array}\right]=\left[\begin{array}{cccc} 1 & r_{12} & \cdots & r_{k1}\\ r_{21} & 1 & \cdots & r_{2k}\\ \vdots & \vdots & \ddots & \vdots \\ r_{k1} & r_{k2} & \cdots & 1 \end{array}\right]$$

where

(3) $$r_{ij}=\frac{\sum_{k=1}^{n}\left(X_{ki}-{\bar{X}}_{i}\right)\;(k_{ij}-{\bar{X}}_{j})}{\sqrt{\sum_{k=1}^{n}{\left(X_{ki}-{\bar{X}}_{i}\right)}^{2}}\sqrt{\sum_{k=1}^{n}{\left(X_{kj}-{\bar{X}}_{j}\right)}^{2}}}$$

Next, the eigenvectors corresponding to the larger p eigenvalues were used to form the projection matrix A.

P-eigenvalues, with a cumulative contribution rate of 95%, were selected for classification purposes.

The new matrix ‘Y’ after the reduction of dimension was obtained (Equation (4)):

(4) Y=XA

#### 2.2.2. k-Means Clustering (Unsupervised Learning)

Clustering is an unsupervised pattern recognition technique used to group data sets into two or more clusters based on similarities and differences noticed between the data points. This study used the k-means clustering method, which clusters data by minimising the sum of squared Euclidean distances from all cluster vectors to their centre [29]. The algorithm is as follows [30]:

1. Sample data sets were defined as $X=\left\{x_{i}|i=1,\;2,\cdots ,n\right\}$,$\;C_{j}\left(j=1,\;2,\cdots ,k\right)$ where *X* denotes the k categories of clusters and $c_{j}\left(j=1,\;2,\cdots ,k\right)$ represents the initial cluster centre. The clusters satisfy:
  a. $C_{i}\neq \emptyset ,i=1,\;2,\cdots ,k$ ;
  b. $C_{i}\cap^{\;}C_{j}=\emptyset ,i,j=1,\;2,\cdots ,k;i\neq j;$
  c. $\overset{k}{\underset{i=1}{\sum }}C_{i}=\left\{x_{1},x_{2},\cdots ,x_{n}\right\}$ .
2. k samples were randomly selected, and $\left(c_{1},c_{2},\cdots ,c_{k}\right)$ was defined as the initial clustering centre.
3. Using the squared Euclidean distance, each sample in the data sets $\left\{x_{i}\right\}$ was assigned to the k cluster centres $c_{i}$.
4. The centre of new cluster $c_{i}\left(i=1,\;2,\cdots ,k\right)$, i.e., $c_{i}=\frac{1}{n}\underset{i=S_{i}}{\sum }x$, where n is the $S_{i}$ cluster domain containing the number of samples, could then be calculated; if $c_{i}\neq c_{i}\left(i=1,\;2,\cdots ,k\right)$, then step (3) was repeated. Otherwise, the algorithm converged, and the analysis ended. Finally, the Silhouette Index (SI) was used to find the optimal cluster number k [31]. As a result, the optimal cluster number k had the highest SI value.

#### 2.2.3. Supervised Classification of AE Signals

AE data from all adhesive and abrasive wear tests were merged to create a library of labelled data for classification using supervised methods. First, hit vectors after a steady state was reached were selected. The merged dataset was then randomly split into two: training and test data at the ratio of 85% to 15%. Three classification models were employed, running each one 20 times. The performance of each model was evaluated using the average classification accuracy, the ratio of correctly predicted cases to a total number of cases and the average F-score (Equation (7)), calculated from the precision (the ratio of the number of correct positive predictions to the total number of positive predictions, Equation (5)) and recall (ratio of correct positive predictions to the number of actual positive cases, Equation (6)), viz. [32]:

(5) $$Precision,\;P=\frac{True\;Positive}{True\;Positive+False\;Positive}$$

(6) $$Recall,\;R=\frac{True\;Positive}{True\;Positive+False\;Negative}$$

(7) $$F-score=\frac{2PR}{P+R}$$

##### Logistic Regression Classifier

Logistic regression, the most common method used in binary classification problems, employs a sigmoid function to compute the probability of a class as a function of the linear combination of multiple variables [33]. The sigmoid function allows mapping real numbers into binary form (0 and 1) and is represented by Equations (8)–(10):

(8) $$g\left(z\right)=\frac{1}{1+e^{-z}}$$

where *z* is an index that combines all the features of X.

(9) $$z=\alpha +\beta_{1}X_{1}+\beta_{2}X_{2}+\cdots +\beta_{k}X_{k}$$

where α and β are unknown constant parameter. Hence, the logistic regression model can be written as:

(10) $$P\left(X\right)=\frac{1}{1+e^{-\left(\alpha +\sum^{\;}\beta_{i}X_{i}\right)}}$$

##### K-Nearest Neighbours (KNN) Classifier

The KNN classifier categorises unknown vectors based on their distance to their nearest neighbours in the training dataset, the distance measure being the weighted squared inverse Euclidean distance between the training set and the unknown vector. The classifier finds the nearest neighbours to the unknown vector and specifies the class with the most representation among those nearest neighbours as the predicted class [29]. The optimum k-number was determined by training with different values and computing the average classification rate. A 5-fold cross-validation method measured classification rates, and the optimum k-number maximised the classification rate whilst minimising training time.

##### Neural Network Classifier

**Figure 2:**
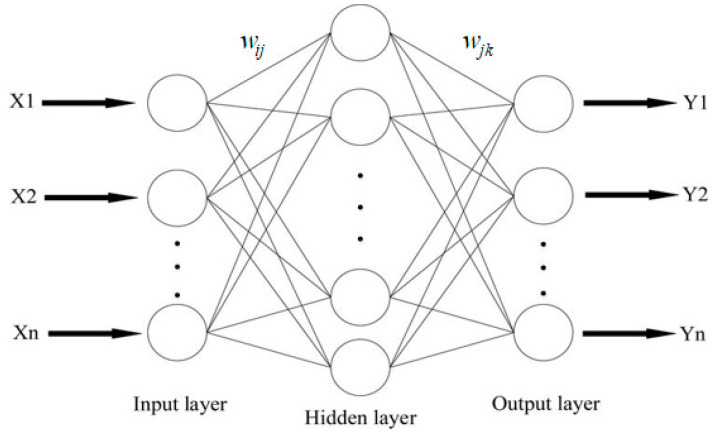
Illustration of a three-layer back propagation neural network.

Finally, back propagation (BP) neural networks, computational architectures modelled after the brain’s architecture [34], were used in the final classification process. The BP network selected was a multi-layer feed-forward neural network with the onward transmission of features and back propagation of errors characteristics of this network. A three-layer pattern recognition feed-forward network comprising one input layer, one hidden layer (10 nodes), and one output layer was used in training (Figure 2). The weights and bias values were updated using the Bayesian regularisation back propagation training function. This training function uses the Levenberg–Marquardt algorithm to optimise the weights and bias [35]. The performance of each iteration was evaluated using a mean square error calculation.

## 3. Results and Discussion

### 3.1. AE Hits and Wear Mechanisms

**Figure 3:**
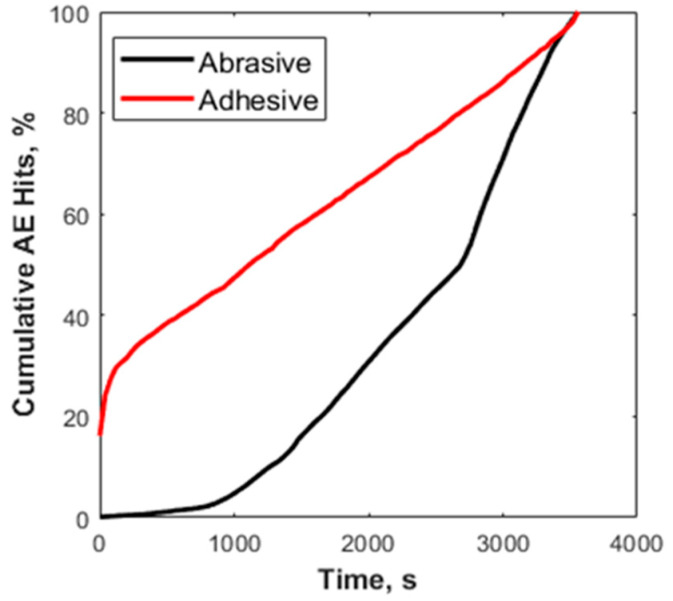
Cumulative AE hits vs time plot for adhesive (red) and abrasive (black) wear tests.

There was a clear distinction among the recorded AE signals between the two wear mechanisms. Hits from the abrasive wear tests were approximately eight times more than those generated during the adhesive tests. The source of these hits is the tribological processes that each specimen is exposed to during testing. In addition to the friction profiles, microscopic examination of worn surfaces provides evidence of several underlying wear mechanisms present only in both tribological tests. These include micro-crack formation and deformation during sliding, and in abrasion, there is evidence of scouring and scratching, resulting in the generation of PEEK wear particles. Further analysis of the frictional data reveals that a quarter of the total AE hits were detected rapidly at the start of the adhesive test. In contrast, it took much longer before 25% of hits were detected (Figure 3) in the abrasive tests.

**Figure 4:**
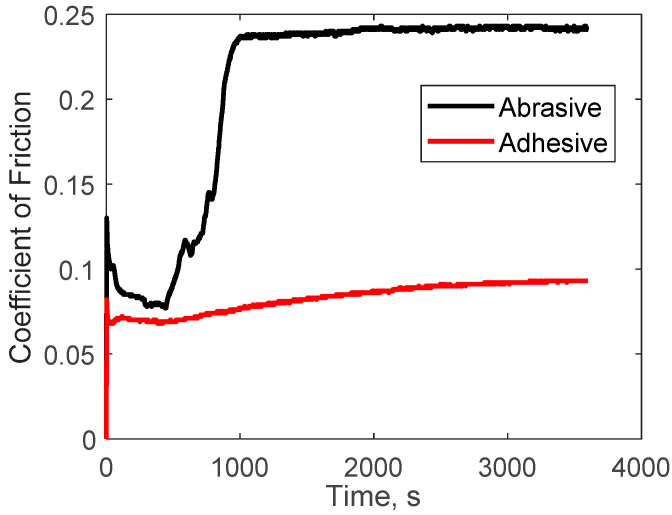
Coefficient of friction vs. time curves for adhesive (red) and abrasive (black) wear tests.

Examination of the friction coefficient curves (Figure 4) provides a better understanding. The initial rapid rise of the friction coefficient in a short period for adhesive tests represents the initial sudden collision of asperities on contacting surfaces. This collision of asperities produces high strain energy, leading to many AE hits. The steady increase in hits correlates with the region of steady friction between contact surfaces, as shown in the friction coefficient (CoF) curve. Therefore, the plot of the cumulative hits for abrasion can also be related to the friction curve. There are three clear stages in the friction curve:

1. Running-in (initial collision of surface asperities and a slight decrease in CoF).
2. A second increase in CoF during prolonged sliding.
3. Steady-state.

These stages explain the three discernible sections in the plot of the cumulative hit.

**Figure 5:**
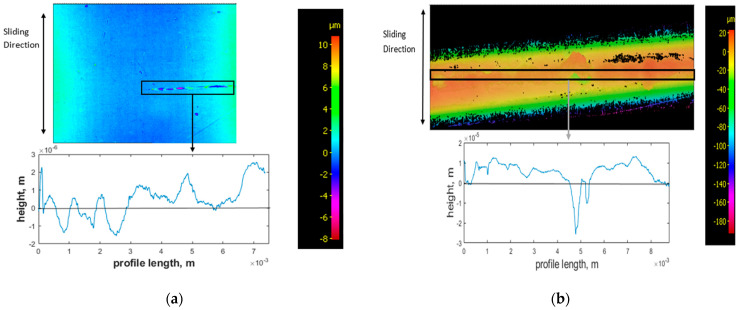
Adhesive test specimens, contour image, and corresponding graphical profiles of worn region after testing for (**a**) steel plate and (**b**) PEEK rods. Regions of high height show wear particle transfer from PEEK to steel plate, indicating adhesion.

An Alicona Infinite focus optical microscope was used to analyse wear scars on the test specimens. Analysis of the wear scar on the steel plate used for the adhesive test (Figure 5a) shows regions of increased height with a corresponding loss of height on the PEEK wear scar surfaces (Figure 5b), indicative of wear particles separating from the PEEK rod and adhering to the metal surface, thereby confirming adhesive wear mechanism.

**Figure 6:**
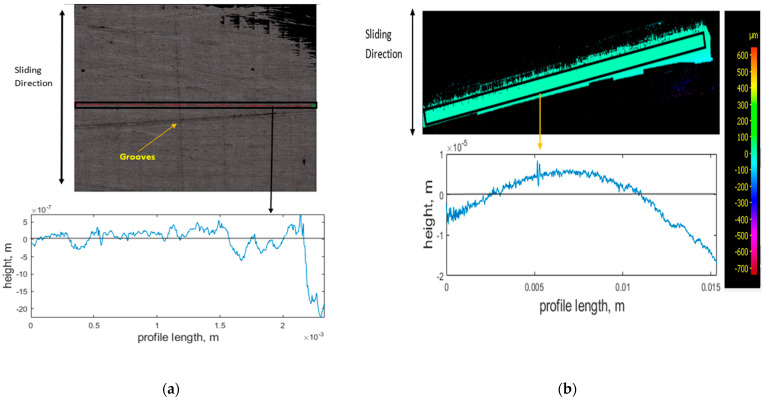
Abrasive test specimens, contour image, and corresponding graphical profiles of the worn regions after testing for (**a**) steel plate and (**b**) PEEK rods. Low topographical height shows material has broken away, indicating abrasive wear mechanism.

The wear scar on the steel plate for the abrasive test (Figure 6a) shows the grooves formed by the pre-test conditioning. The constant low topographical height (around 0 μm) observed in the PEEK and steel’s graphical profiles (Figure 6a,b) indicates that the severity of the asperities has reduced due to material transfer, proving that abrasive wear has occurred. The smooth surface of the PEEK wear scar also implies a complete breakaway of material.

### 3.2. k-Means Clustering

#### 3.2.1. Adhesive Wear

**Figure 7:**
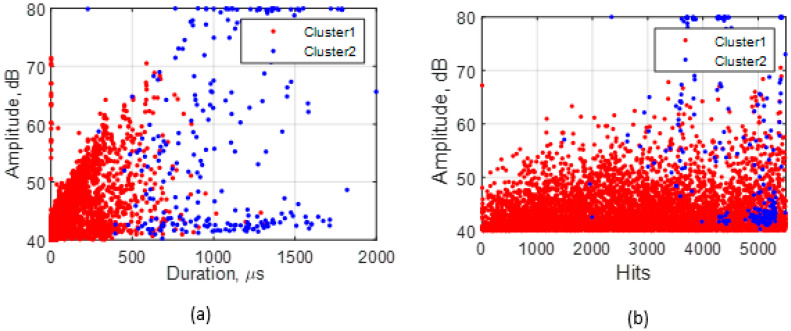
Clustering solutions for adhesive wear test showing (**a**) duration vs amplitude and (**b**) hits vs amplitude plots.

**Figure 8:**
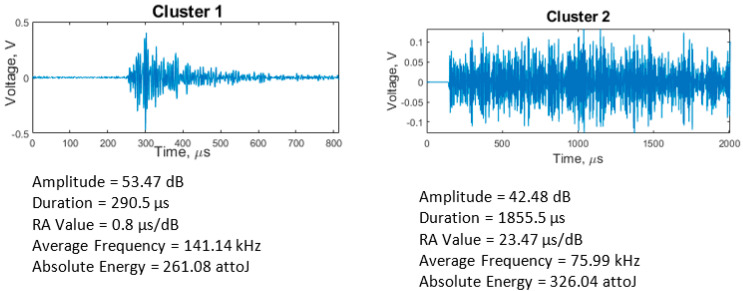
Waveforms of hits in adhesive wear clusters and their features. Cluster 1 event is a burst emission while cluster 2 event is a continuous emission.

According to the Silhouette Index, optimal clustering was obtained with two clusters for the adhesive wear tests. Feature correlation plots are given in Figure 7 for duration vs. amplitude and hits (events) vs. amplitude and show minimum overlapping between clusters. The average values of five AE features for both clusters are shown in Table 3. Events in cluster 2 are of higher intensity than those in cluster 1, evidenced by cluster 2 having higher average values for the features except average frequency for which cluster 1 has a broader range and a higher average. These events indicate the presence of more burst emission types (hits with high amplitude and short duration) in cluster 2 and more continuous emission types (hits with low amplitude and long duration) in cluster 1. The clustering output of all adhesive wear tests showed similar characteristics. A higher percentage of AE events were assigned to cluster 1 (~96%) than cluster 2 (~4%). This increase is expected, as much of the cluster 2 events were generated towards the end of the test. The raw waveforms of sample event from each cluster (Figure 8) also show how events from the two clusters differ.

In general, wear tests tend towards three stages: running-in, steady-state, and severe wear, often followed by rapid failure [36,37]. The running-in stage embodies initial contact between opposing micro-protrusions or asperities on the two contacting surfaces. As the wear process progresses, asperities gradually flatten (the so-called steady-state), and the actual contact area increases, leading to an initial rapid increase in the amount of wear before reducing gradually [36,37,38]. Tests in this study were categorised into running-in and steady-state. Selected experimental conditions meant that it was unlikely that severe wear would be reached due to the short duration of the tests (1 h).

The most likely source of AE hits during running-in emanates from the energy produced during asperity collisions and subsequent junction separation. Other sources could include micro-cracking and material deformation. Due to the high strain energy produced during running-in, the amplitude of the AE hits is high and of short duration (i.e., burst emissions). The steady friction experienced during steady-state should generate mostly continuous emissions (hits with low amplitude and long duration). As observed in Figure 4, the adhesive wear tests reached steady-state very early on in the tests. Reaching steady-state so early in the test makes it challenging to identify a clear distinction between the events recorded during running-in and steady-state. Cluster 1 events shown in Figure 4 were generated for much of the test duration. These AE events combine burst emission (running-in) and continuous emission (synonymous with steady-state tribological conditions). The low amplitude of the AE hits in cluster 1 indicates that most of the signals were generated after asperities have been removed or flattened and the tribological test has reached steady-state conditions. Other sources of AE events in cluster 1 include micro-cracking and fracture of surface asperity junctions. The mean duration value of 85.71 µs is also an indication of burst emissions present from the initial running-in stage. As the sliding progresses further, the continuous contact between surface asperities and the lack of lubricating medium would cause the generation of PEEK wear particles, which then adhere to the steel plate (Figure 5), leading to the emission of higher intensity AE events in cluster 2 compared to cluster 1. A similar observation was made by Hase et al. [36], who found that the generation of wear elements and transfer particles are the sources of burst emissions during adhesive wear tests. The continuous adhesion process would require high strain energy, generating AE hits with high amplitude (burst emissions). As the test nears the 1-h mark, other sources of AE hits would include steady friction between contacting surfaces and crack propagation. These are known to generate mostly continuous emissions; hence, it is expected that hits generated would be mixed emissions (i.e., a combination of burst and continuous). Hence, the duration and RA value of hits in cluster 2 are higher than in cluster 1.

#### 3.2.2. Abrasive Wear

**Figure 9:**
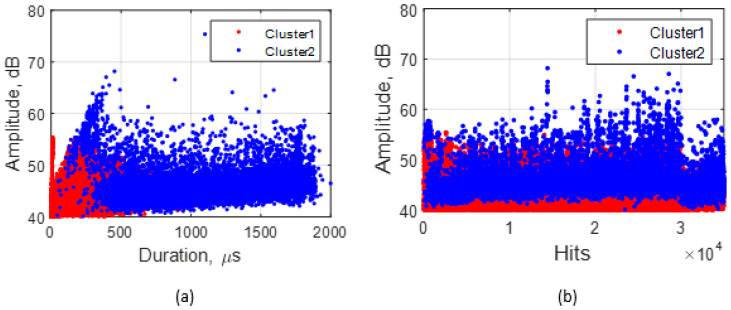
Clustering solutions for abrasive wear showing (**a**) duration vs. amplitude and (**b**) hits vs. amplitude plots.

Optimal clustering was also achieved with two clusters from the abrasive wear tests. Feature correlation plots (Figure 9) show that, unlike in adhesive wear, there is considerable overlap between clusters that can be attributed to the processes of abrasive wear, such as scratching, abrasion, and wear debris generation.

**Figure 10:**
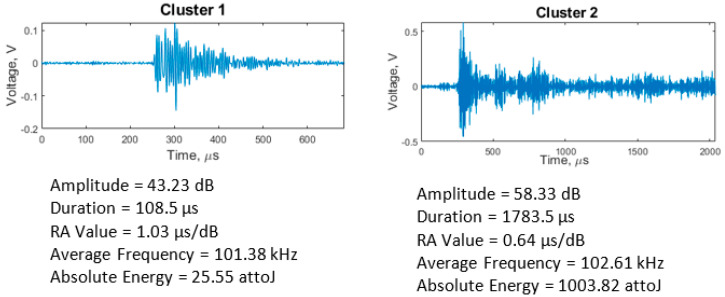
Waveform of abrasive clusters and their features. Event in cluster 1 is a burst emission, while that of cluster 2 is a mixture of continuous and burst emissions.

All abrasive wear tests had similar clustering results (Table 4) to adhesive wear (Table 3), with AE events in cluster 2 having higher intensity than those in cluster 1. Although a higher percentage of AE events were assigned to cluster 1 than in cluster 2, the proportional difference was lower than that observed in the adhesive wear tests (~67% in cluster 1 and ~33% in cluster 2). Therefore, the average amplitude value cannot be relied upon to differentiate between the two clusters. However, the difference between the average values for the two clusters is low (43.76 dB in 1 and 46.22 dB in 2), and both clusters coincided, as shown in the hits vs. amplitude plot (Figure 9b). The raw waveform plots in Figure 10 also show how events from each cluster differ.

Abrasive wear is most commonly of two-body and three-body wear [39]. Figure 4 shows that the running-in stage lasted for c.1000 s, during which time there was a high number of burst emissions, i.e., events of short duration in cluster 1. The source radiated signals will include strain energy produced when surface asperities collide during running-in and the effects of third-body produced during contact [40]. Furthermore, the presence of wear debris increases micro-cutting of the polymer surface, and this, in addition to continued plastic deformation and micro-crack and crack propagation [39], would cause more AE events with burst emission [36], evidenced by the fact that cluster 1 accounts for a higher percentage of total AE events detected.

In addition to the processes mentioned previously, there is friction resulting from the two surfaces sliding against each other, another source of AE events [41,42]. As frictional events are known to generate continuous emissions (events with low amplitude and high duration), it can be concluded that cluster 2 events are primarily due to sliding friction between PEEK and steel. Continuous emissions would also have a long risetime, hence the high RA value for events in cluster 2.

### 3.3. Supervised AE Data Classification

**Figure 11:**
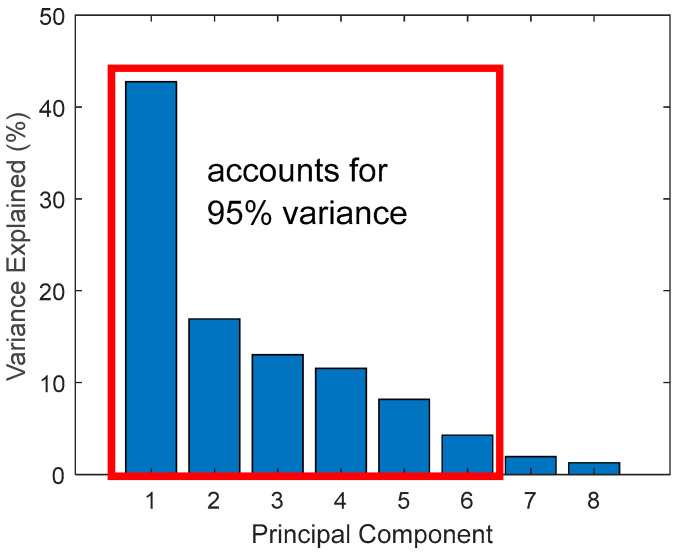
Principal component analysis output.

Hit (vectors) generated during steady-state phases were acquired for supervised classification using the three classification models discussed in Section 2.2.3. After selecting eight features using hierarchical link clustering, PCA extracted six new uncorrelated features that accounted for 95% of the variance between the eight features (Figure 11). After PCA, a 24,075 by 6 input feature matrix corresponding to a 24,075 by 1 labelled output matrix was established for training and testing. Training examples for adhesive wear were labelled ‘0’ and ‘1’ for abrasive wear. Before training, data were randomly split into 85% for training and 15% for testing. The training data were used to train all three models with test data used to evaluate the performance of each model on untrained data. Each classifier was used to train and test 20 times to examine the model robustness.

The average classification accuracy and F-scores for each classifier are presented in Table 5. Since F-scores are calculated assuming that classes are either positive or negative, two F-scores were calculated: adhesive wear as the positive class and abrasive wear as the positive class. An F-score of 1 is indicative of a perfect recall and precision.

All three classifiers have 70% and above accuracy, with logistic regression being the least accurate at 73% (training) and 72% (test) accuracy. However, both the KNN and BP neural network classifiers perform better, with the BP neural network (98%) slightly outperforming KNN (97%). The logistic regression model assumed a simple linear relationship between features, not recognising non-linear patterns, hence the high misclassification rate in comparison to the other two classifiers. By classifying unknown data using distance to its five nearest neighbours, in the case of KNN, all patterns between features are always considered resulting in an optimised classification. In the case of the BP neural network classifier, the use of a training function to optimise the weights and bias values and minimise the squared errors helps train a generalised and optimised model.

The classification accuracy obtained in this study is comparable with other studies that have utilised machine learning algorithms to analyse AE signals. Qiao et al. [18] obtained a 93% average classification accuracy when using a BP neural network to classify 8YSZ thermal barrier coatings according to the indentation failure experienced. In using KNN to classify AE signals acquired during the fatigue testing of carbon fibre composites, Momon et al. [29] obtained an above 90% classification accuracy. McCrory et al. [43] also found that using an artificial neural network can be advantageous for classifying carbon fibre composites according to different damage mechanisms.

**Figure 12:**
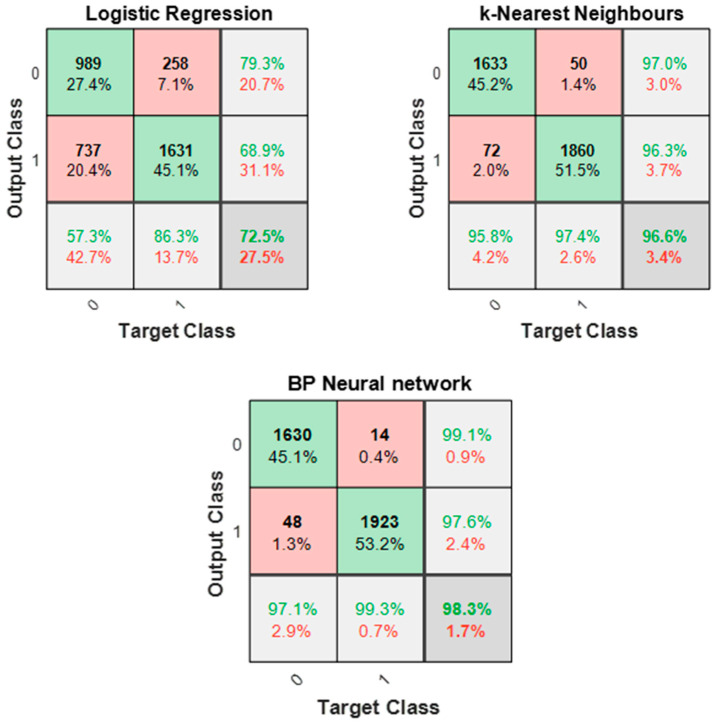
Confusion matrix for all three classifiers.

All three models perform better at recognising abrasive wear features, as indicated by the F-scores (higher when abrasive wear is a positive class) and the confusion matrix plots (Figure 12). Wear processes such as micro-crack, plastic deformation, and sliding friction are common to adhesive and abrasive wear mechanisms, and AE events from these processes are likely to have similar feature characteristics, hence the misclassification. The clustering results further emphasise these similarities between feature characteristics, where overlap can be found in AE feature values in cluster 2 for both wear mechanisms. There are several ways in which the misclassification rate can be further minimised.

- Increase the number of neurons in the hidden layer for the BP neural network [44].
- Increase features used for classification [45] by choosing more features or deriving new features through the mathematical combination of original features. More features can help improve the model by recognising more patterns between features and improving the classification accuracy.
- Increase the number of training examples. Having more examples would help the model learn better, thereby improving classification accuracy.

KNN classifiers do not learn from the training data. Instead, training data are used to classify test data. Although the KNN classifier has a high classification accuracy, increasing training examples and/or features would increase the time it takes for new data to be classified. Additionally, finding the optimised k-value can be time-consuming, and considering that the optimal k-value will be based on input features, the process would have to be repeated every time training data are updated [46]. The BP neural network has an advantage over the KNN classifier as it is unnecessary to learn the details of functions used in hidden layers. It is relatively simple to train with new data. Moreover, since the neural network learns from the training data, it is acceptable for the training time to be high since the test time is unlikely to be high [47]. The ever-changing nature of AE signals would require a classification model that is simple and easy to manipulate, making the neural network classifier the best choice.

## 4. Conclusions

Pattern recognition techniques have classified AE signals from tribological tests acquired under simulated artificial joint conditions. Using k-means clustering, AE hits detected during a tribo-acoustic test based on the different tribological processes have been grouped, and subsequently diagnosing different stages of tribo-acoustic tests using similarities and differences between AE features.

In this study, principal component analysis was used to derive new uncorrelated features before classification using logistic regression, KNN, and BP neural networks. Although both KNN and BP neural networks had a classification accuracy of above 95%, we concluded that the BP neural network is the optimum choice of pattern recognition technology due to its simplicity and dynamism.

The high classification accuracy shows that features of AE signals acquired under controlled joint conditions have hidden relationships that can be identified using pattern recognition techniques. This finding is clinically significant because it shows that the potential for using AE to interpret biotribological mechanisms from artificial and natural joints can also be used to differentiate between wear mechanisms, an excellent step towards AE being used as a joint pathology diagnostic tool.

## Figures and Tables

**Table 1:** TE77 and AE acquisition parameters.

	Parameters	Value
**TE77 TEST PARAMETERS**	Load	150 N
	Frequency	2 Hz
	Stroke	12.5 mm
	Test Duration	1 h
	Lubricant	Dry (to induce adhesive wear) and Ringer’s Solution (to induce abrasive wear)
**AE ACQUISITION PARAMETERS**	Threshold	40 dB
	Pre-amplifier gain	60 dB
	Band Pass filter	100–600 kHz
	Sampling rate	2 MHz.

**Table 2:** Selected AE features (after hierarchical clustering) and their definitions.

No.	AE Features	Definition
1	Amplitude	Maximum amplitude of the signal.
2	Duration	The time from first threshold crossing to the last threshold crossing.
3	Counts to Peak	Number of crossings from first crossing to the point where maximum amplitude is reached.
4	RA Value	Time per amplitude needed for signal to reach its peak value. Expressed as ratio of risetime to amplitude.
5	Average Frequency	Signal counts over signal duration.
6	Peak Frequency	Frequency corresponding to the peak value of the power spectrum of the FFT transform.
7	AE Root Mean Square	Root mean square of voltage curve.
8	Absolute Energy	The true energy of the signal on a 10 kohm resistor.

**Table 3:** Range and mean (including standard deviation) of five features per cluster for adhesive wear tests.

AE Features	Cluster 1		Cluster 2	
	Range	Mean (*std*)	Range	Mean (*std*)
Amplitude, dB	40–71.43	43.81 (*4.30*)	40.27–79.99	56.70 (*14.67*)
Duration, µs	0.5–1308	85.71 *(133.16*)	226.5–1998.5	1048.60 (*347.05*)
RA Value, µs/dB	0–12.47	0.57 (*1.27*)	0.01–35.25	10.08 (*7.84*)
Average Frequency, kHz	0–1000	333.73 *(378.23*)	1.46–194.26	60.36 *(54.67*)
Absolute Energy, attoJ	0.26–28,610	96.48 (*648.62*)	41.29–687,510	59,834 (*1.20 × 10^9^*)

**Table 4:** Range and mean (including standard deviation) values of five features per cluster for abrasive wear test.

AE Features	Cluster 1		Cluster 2	
	Range	Mean (*std*)	Range	Mean (*std*)
Amplitude, dB	40.01–55.42	43.76 (*2.80*)	40.14–75.36	46.22 (*3.28*)
Duration, µs	0.5–1200	116.84 (*157.93*)	45.5–2010	1060 (*422.05*)
RA Value, µs/dB	0.01–14.42	0.87 (*1.75*)	0.01–42.55	13.54 (*9.40*)
Average Frequency, kHz	0–2000	212.22 (*298.36*)	1.57–193.80	27.72 (*23.44*)
Absolute Energy, attoJ	0.57–567.11	45.99 (*58.10*)	11.73–30,200	326.96 (*472.03*)

**Table 5:** Summary of classifiers’ performance.

CLASSIFIER	Average Training Accuracy (±std)	Average Test Accuracy (±std)	Average F-Score (±std)	
			Adhesive is positive	Abrasive is positive
Logistic Regression	0.73 (*±0.0025*)	0.72 (*±0.0048*)	0.66 (*±0.0096*)	0.77 (*±0.0036*)
Weighted k-Nearest Neighbours	0.96 (*±0.0007*)	0.97 (*±0.0026*)	0.96 (*±0.0029*)	0.97 (*±0.0025*)
Back Propagation Neural Network	0.98 (*±0.0014*)	0.98 (*±0.0024*)	0.98 (*±0.0024*)	0.98 (*±0.0023*)

## Data Availability

Data available on request from the corresponding author.
